# The Impact of Impulsive Traffic on Cellular Internet of Things Network Performance Indicators

**DOI:** 10.3390/s24010046

**Published:** 2023-12-21

**Authors:** Tammam Zuhair Dawood, Mikhail Sergeevich Stepanov, Matvey Kudashkin, Arina Shaimardanova, Petr Lapko

**Affiliations:** 1 Faculty of Communication Networks and Systems, Moscow Technical University of Communications and Informatics, 8A, Aviamotornaya Str., 111024 Moscow, Russia; m.s.stepanov@mtuci.ru (M.S.S.); kudashkin.matvey@mail.ru (M.K.); arina_sh@bk.ru (A.S.); petyalapko@mail.ru (P.L.); 2 Faculty of Mechanical and Electrical Engineering, Tishreen University, Latakia P.O. Box 2237, Syria

**Keywords:** NB-IoT, LTE, LPWAN, licensed spectrum, recursive algorithms, mathematical modeling

## Abstract

The use of wireless sensor networks and the Internet of Things has increased dramatically in the last decade. The sensors measure the required parameters and send them to the data processing centers using one of the various wireless transmission technologies (often using cellular infrastructure) to make the appropriate decision. Files containing measurement information can arrive in bursts simultaneously, which is a critical issue to be aware of. The purpose of this work is to develop and analyze a model to evaluate the effectiveness of an LTE (Long-Term Evolution) cell in serving requests from NB-IoT (Narrowband Internet of things) devices when these requests are received in bursts rather than individually. In the article, the common uses of the Internet of Things in our modern era were discussed, the NB-IoT technology was paid attention to, and a mathematical model to evaluate the performance of an LTE cell in the case of impulsive arrivals of NB-IoT requests was built. Finally, the computational algorithm and numerical evaluation were introduced.

## 1. Introduction

The Internet of Things (IoT) is a concept that involves connecting various physical objects to the internet that are not typically associated with it, such as appliances, cars, buildings, and so on. This allows them to exchange data and interact with each other without human involvement. The relevance of the Internet of Things today lies in the fact that it enables the improvement of productivity, safety, energy efficiency, and user convenience in many systems and devices. Nowadays, IoT is becoming an increasingly relevant area of technological development.

There are many examples of the application of the Internet of Things in our current era, including but not limited to smart homes, smart transportation, smart industry, and smart agriculture [1,2,3,4,5,6,7].

Thus, the Internet of Things is a relevant direction in the modern world that allows for increasing efficiency, safety, and convenience in using many systems and devices in various areas of activity.

LPWAN (Low Power Wide Area Network) technologies are considered one of the fastest-growing technologies among the Internet of Things technologies today due to their provision of cheap, long-range solutions with efficient energy consumption [8,9].

Low-Power Wide-Area (LPWA) refers to a set of wireless communication technologies specifically crafted to facilitate the establishment of wireless sensor networks. LPWAN technologies can be classified according to the frequency spectrum on which they operate into two types (Table 1): The first type operates within the licensed frequency spectrum (cellular spectrum), which makes it secure with low interference. The second type is the type that uses an unlicensed frequency spectrum, which leads to high interference potential.

**Figure 1:**
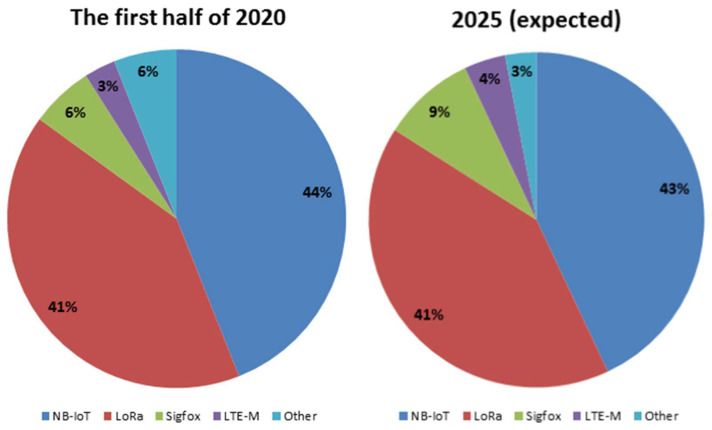
Share of LPWA connections worldwide [11].

NB-IoT is the most widely used LPWA technology, followed by LoRaWAN, Sigfox, and other technologies. Therefore, studying the performance of NB-IoT networks is very important. In Figure 1, the global share of LPWA connections in the first half of 2020 and 2025 is shown.

In this paper, we focus on studying how the performance indicators of cellular networks change in the case of impulsive traffic, which occurs when a huge number of requests arrive (or a huge number of terminals connect) simultaneously. This is a situation that occurs widely in cellular Internet of Things (CIoT) networks for several reasons, including: (1) Random deployment of sensor nodes in a geographical area may cause the presence of a large number of sensors in the same cell at the same moment. These sensors may generate and send their own traffic simultaneously, resulting in impulsive traffic generation. (2) Using hubs and concentrators as an intermediary between measuring devices and the base station may lead to an instantaneous, impulsive arrival.

The article is organized as follows: related works are reviewed. The NB-IoT technology is discussed. The mathematical model built in order to study the performance indicators of CIoT networks with impulsive incoming requests is explained. Numerical experiments are carried out to evaluate the model, and then our results are reviewed. Finally, conclusions are drawn.

## 2. Related Works

Narrowband Internet of Things (NB-IoT) is a type of wireless communication developed particularly for the Internet of Things. It is low-power, wide-area technology that enables a wide range of devices to be connected to the internet. It is designed to provide reliable and secure communication over long distances while minimizing power consumption and operating costs. This technology has emerged as a popular choice for connecting a variety of IoT devices, including smart meters, environmental sensors, and asset trackers.

NB-IoT has received great attention in recent years, and the focus has increased by researchers on analyzing the performance of this technology, the possibility of implementing it in different conditions of the cellular network, and the challenges faced by the deployment of this technology.

The authors in [9,12] analyze NB-IoT technology since its inception and the improvements that were introduced in successive releases from Rel. 13 to Rel. 16. They also study the integration of this technology with different cellular technologies, such as 5G, non-orthogonal multiple access (NOMA), etc.

In [13], the authors study NB-IoT and eMTC (Enhanced Machine Type Communications) technologies, evaluate their performance (using the NS3 simulator) when implementing smart city solutions, and compare these two technologies in terms of the number of devices that can be served and their energy consumption.

The performance of NB-IoT technology in 5G heterogeneous wireless networks (HetNet) was studied in Ref. [14], and according to the results obtained by the authors, a significant performance deterioration was observed in this deployment scenario.

The authors in [15] were interested in studying the performance of NB-IoT when implemented based on 5G technology via the Geostationary Equatorial Orbit and concluded that it is necessary to adjust the physical channels of NB-IoT to adapt to the type of service required. Here, the authors used Python for modeling and performance evaluation, while in our research, we will model using MATLAB the implementation of NB-IoT solutions using 4G terrestrial networks, which have a lower communication delay than non-terrestrial networks (NTN).

In [16], the impact of coverage extension on the performance of NB-IoT terminals and the limitations that this event imposes on battery and communication delays were studied.

The experimental results reached by researchers in Ref. [17] after evaluating the performance of NB-IoT technology on the Belgian Orange network prove that NB-IoT is a reliable, secure, available, and scalable technology with low cost and energy consumption, which explains the importance of our study of this promising technology.

The authors in [18] proposed an improved strategy to schedule NB-IoT terminal servicing according to priority, and this strategy achieved a reduction in network congestion. The authors in this paper modeled the arrival of requests as following a uniform distribution and beta distribution, while the arrival of requests may be a random impulsive arrival, and this limits the network performance.

In [19], the authors study the effect of changing transmission distance and the presence of obstacles on the performance of smart systems based on NB-IoT.

In addition, there has been a great research effort directed at evaluating the performance of NB-IoT in specific use cases, and this technology has been proven to be good for use in emergency situations such as remote patient monitoring [20] and monitoring power grid parameters [21].

On the other hand, a section of researchers [22,23] opt for comparing unlicensed LPWAN technologies with cellular LPWAN technologies (including NB-IoT) in an attempt to find and identify the strengths and weaknesses, pros and cons, applications and use cases, and limits for each of these technologies.

Our contribution in this article is to discuss the case of group reception of resource requests in NB-IoT networks and highlight the importance of studying this case when planning radio resources because neglecting it may lead to network performance degradation.

A summary of related works is presented in Table 2.

The objective of our study is to determine whether the impulsive nature of cellular IoT has an impact on the throughput of these networks and to evaluate the network performance in this case.

Research hypotheses to be proven:

**Hypothesis 1.**  *The failure to take into account the impulsive nature of incoming traffic leads to the fact that the throughput is planned incorrectly (less than it should be), which leads to increased loss.*

**Hypothesis 2.**  *The greater the number of incoming requests at the same time, the worse the system’s performance becomes.*

## 3. NB-IoT

The development of NB-IoT started as a part of the 3rd Generation Partnership Project (3GPP) Release 13. The objective was to create a technology that could operate in the unused spectrum of existing LTE networks without causing any interference. The first version of the standard, known as Release 13 Narrowband, was published in June 2016 [12].

NB-IoT operates in the licensed spectrum and can be deployed in various frequency bands, including the (450, 800, 900, 1800, and 1900–2690) MHz bands. The device’s receive bandwidth is 180–200 kHz. The frequency band used for NB-IoT deployment depends on regional regulations and the availability of spectrum. The 900 MHz band is the most used frequency band for NB-IoT deployment in Europe and Asia, while the 800 MHz band is widely used in North America. However, the use of NB-IoT may vary depending on the region. These differences in deployment may affect the coverage, reliability, and cost of NB-IoT networks. In addition, different regulatory requirements may apply in different regions, which may affect the deployment and operation of NB-IoT networks. Nevertheless, NB-IoT is projected to play an important role in the IoT ecosystem, allowing the creation of novel IoT applications all over the world.

NB-IoT uses orthogonal frequency division multiplexing (OFDM) for data transmission. OFDM is a modulation technique that divides the available spectrum into multiple subcarriers, each carrying a separate data stream. The subcarriers are orthogonal to each other, which means that they do not interfere with each other. This enables NB-IoT to transmit data reliably even in environments with high levels of interference. In addition to OFDM, NB-IoT also uses Differential Quadrature Phase Shift Keying (DQPSK) modulation for control signaling.

**Figure 2:**
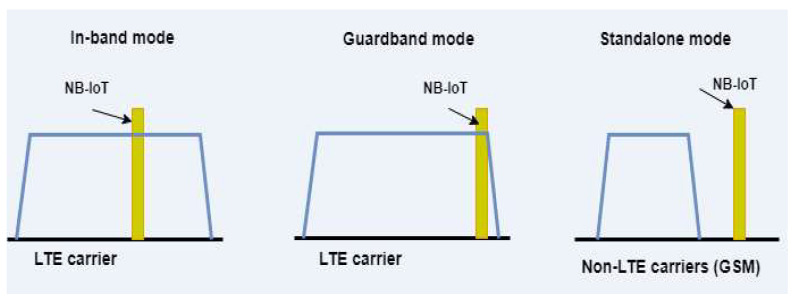
NB-IoT operation modes [9].

The 3GPP Release 13 standard defines three deployment modes for NB-IoT, each with a different channel placement method (Figure 2). In-band mode utilizes the same LTE carrier for both data and control signaling, with NB-IoT channels placed within the LTE carrier. Standalone mode operates independently of the LTE network and uses a separate carrier for NB-IoT data transmission. Guard band mode uses the unused spectrum between two LTE carriers for NB-IoT data transmission. The data transfer rate of the technology is 250 Kbit/s, the coverage level is 164 dB, and the power class is 20–23 dB [24].

**Figure 3:**
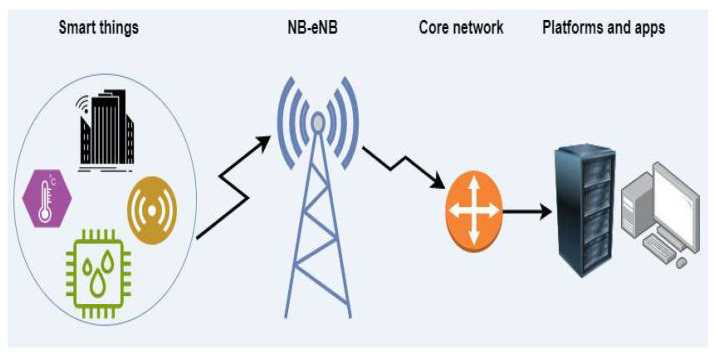
NB-IoT network architecture [25,26,27].

NB-IoT utilizes a simplified network architecture compared to traditional LTE networks. The NB-IoT network consists of three main components (Figure 3): the user equipment (UE), the NB-IoT base station (NB-eNB), and the core network (CN). The UE is the device that sends and receives data over the network. The NB-eNB is the base station that communicates with the UE and the CN. The CN is responsible for managing the network and routing data to its final destination.

**Figure 4:**
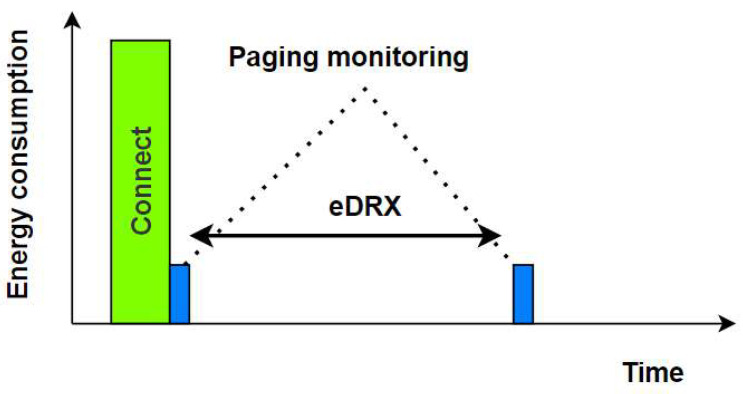
eDRX mechanism [26].

The key feature of NB-IoT is its low consumption of power, which allows devices to operate for several years on a single battery or even without a battery, relying on energy harvested from the environment. This feature can be obtained as a result of PSM and eDRX technologies. (1) The principle of PSM technology is to transfer the device to the PSM state after remaining for a specified period of time in the IDLE state. It is a new state that has been added in 3GPP Release-12 to the two states that the device can be in (Idle and Connect). In this state, the radio unit is completely turned off until it has messages to send, then the radio unit is restarted [26]. (2) The eDRX was proposed in Release 13 to improve power consumption by increasing the paging monitoring interval. The eDRX mechanism is shown in Figure 4.

Another important feature is its ability to operate in harsh environments, such as underground areas, where other wireless technologies may not be able to operate. This makes NB-IoT ideal for various IoT applications, including smart cities, smart agriculture, and industrial automation [24].

NB-IoT is renowned for its superior building penetration capabilities, enabling it to reach devices located deep indoors or underground. The signal propagation of NB-IoT is particularly effective due to its narrowband nature, which allows signals to penetrate obstacles more efficiently than wideband signals. However, it has limited non-line-of-sight (non-LoS) propagation due to the high frequency it operates on [28].

NB-IoT is compatible with LTE, meaning it is able to work within it, but there are some differences between these two technologies. NB-IoT operates in FDD (Frequency-division duplexing) half-duplex mode, while LTE supports full-duplex. NB-IoT has its own physical channels and signals, which are listed in Table 3 [29].

NB-IoT technology has several uses, including [12]:

- Smart Metering via NB-IoT: This technology automates utility meter readings, decreasing manual labor and boosting accuracy.
- Animal Tracking with NB-IoT: Allows for real-time tracking of livestock or animals, enhancing animal care and management.
- NB-IoT Smart Lighting: Enables remote control and monitoring of lighting systems, resulting in increased energy efficiency.
- NB-IoT Facility Management: Improves operational efficiency by facilitating the monitoring and management of building systems.
- NB-IoT Environmental Monitoring: Aids in the collection of environmental data, which aids in climate study and conservation initiatives.
- NB-IoT garbage management: automates garbage level monitoring in bins.

NB-IoT is a promising wireless technology that has emerged as a popular choice for IoT applications. Its low power consumption, reliable connectivity, and ability to operate in harsh environments make it an ideal choice for various IoT applications. As the demand for IoT devices continues to grow, NB-IoT is projected to become increasingly essential in the coming years.

## 4. Materials and Methods

Teletraffic theory plays a vital role in understanding and managing the behavior of traffic flows within telecommunication networks. By utilizing various models and concepts, network operators can accurately predict performance metrics, optimize resource allocation, and enhance the quality of service provided to users. As communication technologies continue to evolve, teletraffic theory will remain a fundamental tool for network planning, optimization, and efficient resource management [30,31,32,33,34,35].

In this work, teletraffic theory was mainly relied upon to build the mathematical model, which will be explained in detail later in the following paragraphs. The recursive method was also used to find the output and results of the proposed mathematical model.

MATLAB was used as a working environment to simulate the mathematical model and obtain results.

Abbreviations used in this manuscript together with their meanings are presented in Table 4.

## 5. Mathematical Model

In the cellular Internet of Things, cellular network operators use their infrastructure to transfer the data of devices (things) along with the data of cellular users. This necessarily requires finding an effective way to share resources between these things and users so that the quality of service is guaranteed to be maintained for both types. The most widely used cellular IoT technology is NB-IoT. The 3GPP proposed the in-band deployment pattern in an attempt to solve the problem of resource sharing between LTE and NB-IoT devices, but no specific method has been defined in order to allocate the resources. When allocating resources between these two types of users, it is important to study the traffic characteristics of each type in order for the solutions applied to be more beneficial and achieve a higher quality of service for each of them.

The time intervals between successive arrivals of requests in the Erlang model have an exponential distribution. Because the appearance of requests is random, they may appear more frequently at some intervals and less frequently at others. However, this property of the Poisson flow is insufficient to reflect the impulsive nature of request receipt, which is inherent in the operation of some communication systems. The pulsations caused by the appearance of requests for information transmission resources range in character. These can be concentrations that reflect the specifics of the formation of a stream of requests for the transmission of real-time service traffic or the characteristics of the grouping of data traffic information messages.

In CIoT scenarios, the sensors measure the required parameters and send them in a session series using cellular infrastructure to the data processing centers to make the appropriate decision. These sensors use concentrators and relays to accumulate and amplify signals. So that files containing measurement information can arrive in bursts simultaneously, this is a critical issue to be aware of.

Let us construct a mathematical model describing the allocation of channel resources, considering the impulsive character of request arrival. Unlike the Erlang model, we offer a two-level approach for reproducing the incoming traffic flow here.

**Figure 5:**
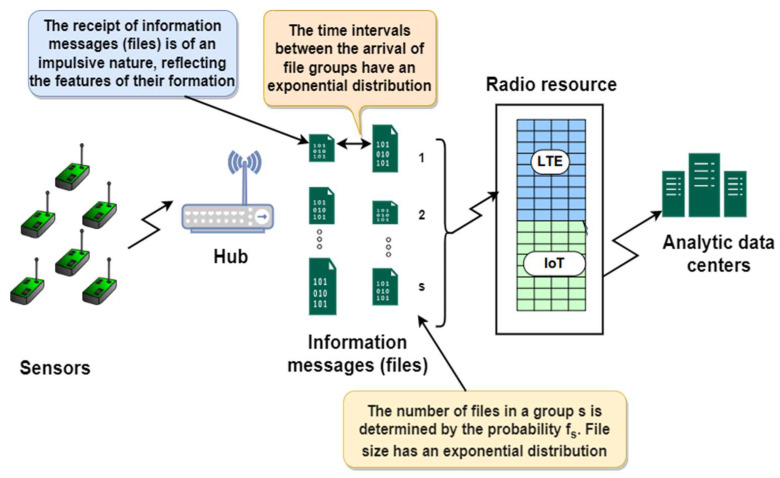
The analytical model of an LTE cell simultaneously serving IoT users as well as cellular users, considering the impulsive nature of IoT requests [author’s own processing].

The analytical model of an LTE cell simultaneously serving IoT users as well as cellular users, considering the impulsive nature of IoT requests, is shown in Figure 5.

Let υ denote the total number of resource units that the LTE cell can provide to IoT devices. The arrival of a group of requests obeys the Poissonian model, with group arrival intensity denoted by λ. The time required to serve one request is exponentially distributed, with a mean duration equal to μ. Each incoming group can contain a different number of requests that is not equal to zero; the probability that the group has s requests is equal to $f_{s}$, where the count of files in the group *s* = 1, 2,…, *n*. For convenience, let’s take *n* = *υ*, so s will vary from 1 to υ. Easily, we could know that $\sum_{s=1}^{\upsilon }f_{s}=1$.

The average amount of requests in the group is denoted by b and can be calculated by the following equation:

(1) $$b=\sum_{s=1}^{\upsilon }s.f_{s},$$

When a group of requests is received, one of three scenarios is possible:

1. There is sufficient free resource on the chosen resource (slice) to service the whole group. Then, this group will be served at the same time.
2. The slice lacks sufficient free resources to satisfy the whole group. In this situation, a partial group of the whole group will be served, while the rest will be lost and not renewed.
3. The resources are completely busy. Then no request will be served, and the whole group will be lost without being revived.

Let the variance in the total amount of requests for a given incoming group be denoted by $\sigma^{2}$.

As the number of requests in the group moves away from its middle-value b, the value of the variance $\sigma^{2}$ will increase, and this leads to an increase in the impulsiveness of the incoming requests.

(2) $$\sigma^{2}=\overset{\upsilon }{\underset{i=1}{\sum }}f_{i}{\left(i-b\right)}^{2}$$

Indicate the model under consideration’s state space with $S=\left\{\left(i\right),\;i=0,1,\ldots ,\upsilon \right\}$. A random process $r\left(t\right)=i\left(t\right)$ explains their changes over time, where $i\left(t\right)$ denotes the number of channels utilized to handle requests at a given point in time t. The process may be categorized as a Markov process since the model’s random variables all have an exponential distribution and are independent of other variables.

Denote by $p\left(i\right)$ and P(i) the normalized and unnormalized probability that the model has i channel in the busy state, respectively. To find these probabilities, a set of equilibrium equations must be created and solved. The usual approach of teletraffic theory is used to tackle this problem. We obtain the following equation system:

(3) $$\begin{array}{c} P\left(0\right)\lambda =P\left(1\right)\mu \;;\;\;i=0\\ P\left(i\right)\left(\lambda +i\mu \right)=\left(P\left(0\right)f_{i}+P\left(1\right)f_{i-1}+\ldots +P\left(i-1\right)f_{1}\right)\lambda +P\left(i+1\right)\left(i+1\right)\mu \;;\;\;i=1,\ldots ,v-1\;\\ P\left(v\right)v\mu =\left(P\left(0\right)f_{v}+P\left(1\right)\left(f_{v-1}+f_{v}\right)+\ldots +P\left(v-1\right)\left(f_{1}+f_{2}+\ldots +f_{v}\right)\right)\lambda ;\;\;i=v \end{array}$$

A recurring relation relating $P\left(j\right)$ can be obtained via algebraic manipulations of the system of equilibrium equations. It appears as follows:

(4) $$j\mu P\left(j\right)=\lambda \left(P(0)\sum_{i=j}^{\upsilon }f_{i}+P(1)\sum_{i=j-1}^{\upsilon }f_{i}+\ldots +P(j-1)\sum_{i=1}^{\upsilon }f_{i}\right)$$

The quality of service of the model is controlled by these indicators:

- m—the average number of occupied channels.
- $\pi_{c}$—the ratio of lost requests.
- $\pi_{t}$—the portion of time when all channels are busy.

The following are the stages in the algorithm for determining $p\left(i\right)$:

1. Set the value of the unnormalized probability in the case of empty channels (no busy channels) equal to 1, and we can write it as $P\left(0\right)=1$.
2. Find the unnormalized probabilities P(j) for *j* = 1,…, *υ* using the recursion in Equation (4).
3. Calculate the normalization constant *N*:
  (5) $$N=\sum_{j=0}^{\upsilon }P(j)$$
4. Find the normalized probabilities as follows:
  (6) $$p\left(j\right)=\frac{P\left(j\right)}{N},\;\;\;j=0,1,\ldots ,\upsilon$$
5. Calculate the quality of service indicators as follows:
  (7) $$\pi_{c}=\frac{1}{b}\overset{\upsilon }{\underset{i=1}{\sum }}p\left(i\right)\sum_{k=0}^{i-1}f_{\upsilon -k}.(i-k)$$
  (8) $$\pi_{t}=p\left(\upsilon \right)$$
  (9) $$m=\overset{\upsilon }{\underset{i=0}{\sum }}p\left(i\right)i$$

## 6. Numerical Assessment

Let us consider an LTE network cell serving an area containing a large number of telemetry devices that send their measured values in the form of files. The file arrival model is a batch model, with its parameters determined as follows:

- Set file QoS to $\pi_{c}=0.05$.
- The transmitted file’s size is distributed exponentially, with a mean value of F = 100 bytes.
- The information transmission rate *u* given by one virtual channel utilized to service the IoT device’s application will be considered to be u=100 kbps.
- The file transfer rate *C* that can be offered by the system can be calculated by the relation: C=υ*u.
- The time required to serve one file is exponentially distributed with a mean duration equal to *μ*, where: μ=u/F.
- The potential load of files is set at a=15 Erl.
- The relationship λ=aμ/b yields the parameter of the Poissonian distribution of the group arrival intensity.

Consider the following three approaches to file distribution in an incoming group:

1. First case: each incoming group can contain only one file with a probability equal to $f_{1}=1$. In this case, $b=1,\sigma^{2}=0$;
2. Second case: the incoming group can contain several files, which can be equal to 1, 2, …, 15, with the same probability for each one $f_{i}=1/15;i=1,2,\ldots ,15$. In this case, $b=8,\sigma^{2}=18.6667$;
3. Third case: incoming groups can contain only 1 or 15 files with the same probability for both $f_{1}=f_{15}=0.5$. In that case, $b=8,\sigma^{2}=49$.

## 7. Results

In this section, we will present the results of applying our mathematical model to the cases under study (mentioned in Section 6) and compare the quality of service in each of the three considered cases.

**Figure 6:**
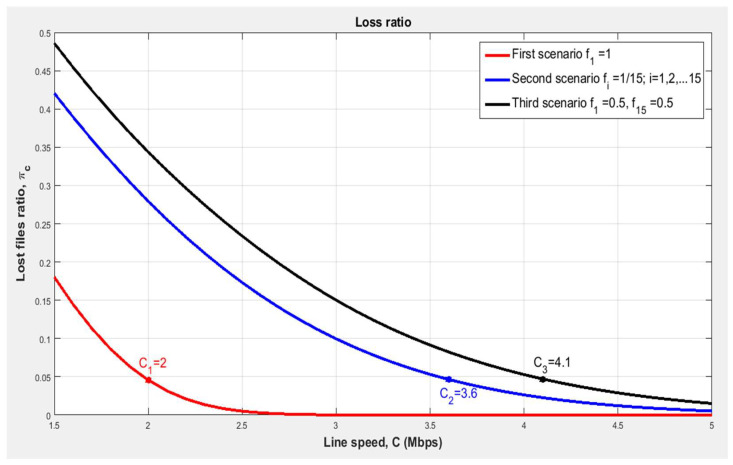
Dependence of the proportion of lost files on the file transfer speed provided by the allocated resource and the dispersion of the amount of group files [author’s own processing].

Figure 6 shows the dependence of the proportion of lost files on the file transfer speed provided by the allocated resource and the dispersion of the number of group files. We notice that with an increase in the dispersion of files’ amount in the group $\sigma^{2}$, the required resource increases to obtain the same required quality of service $\pi_{c}=0.05$. For example, to obtain a loss ratio of 0.05 in the normal case (first case) without impulsive file receiving (the dispersion of the number of group files $\sigma^{2}=0$), a line speed equal to C_1_ = 2 Mbps is needed. In the second case, with a dispersion equal to $\sigma^{2}=18.6667$, more line speed is needed (C_2_ = 3.6 Mbps). In the third case (the worst case), with the highest dispersion of the number of files in the group $\sigma^{2}=49$, it is clear that the required line speed to obtain the same loss ratio ($\pi_{c}=0.05$) is equal to C_3_ = 4.1 Mbps, which is double the value of the line speed required in the normal case.

**Figure 7:**
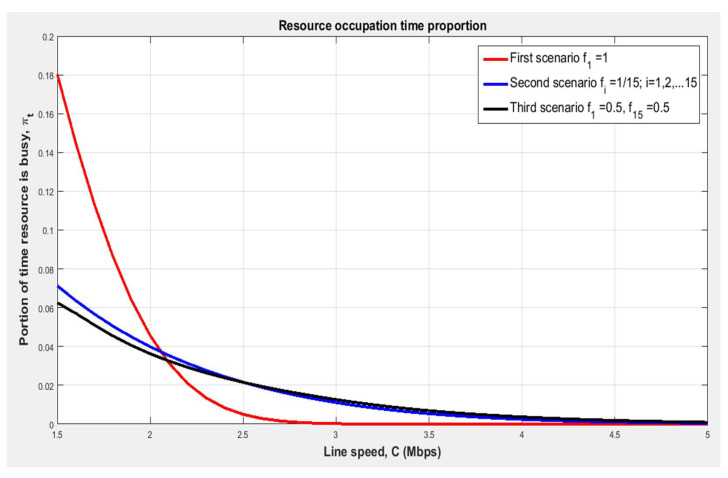
Dependence of the time proportion of resource occupancy on the file transfer speed provided by the allocated resource and the variance of the amount of group files [author’s own processing].

Figure 7 shows the dependence of the proportion of time on the file transfer speed provided by the allocated resource and the dispersion of the number of group files. We notice that with the increase in the line speed C, the proportion of resource occupancy time $\pi_{t}$ decreases, but the greater the variance in the number of group files $\sigma^{2}$, the more file transfer speed C is required to obtain the same required share of resource occupancy time $\pi_{t}$.

**Figure 8:**
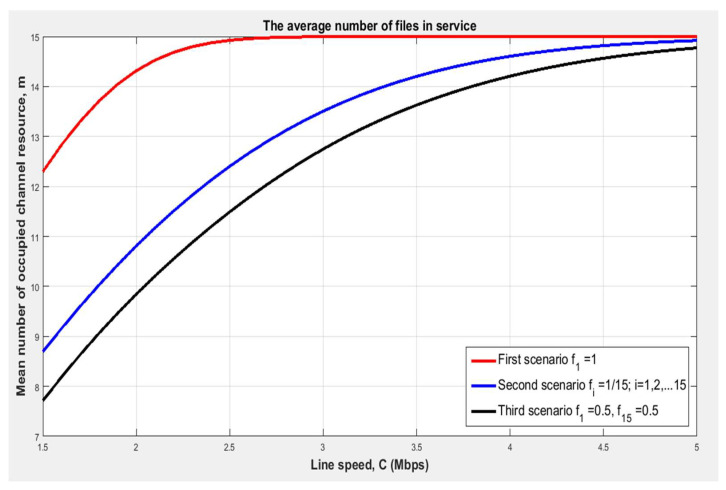
Dependence of the average file servicing on the file transfer speed provided by the allocated resource and the dispersion of the amount of group files [author’s own processing].

Figure 8 shows the dependence of the proportion of average file servicing on the file transfer speed provided by the allocated resource and the dispersion of the number of group files. We notice that with an increase in the variance in the amount of group files $\sigma^{2}$, the average number of busy channels m (serving rate) significantly decreases. For example, in the first scenario, a line speed equal to C_1_ = 2.5 Mbps is needed to obtain an average of 15 files in service (m = 15 files), while in the third scenario (impulsive case), a greater line speed is needed (C_3_ > 5 Mbps) to obtain the same average number of files in service (m = 15 files).

From the above discussion, the values of the indicators under study are affected by the impulsive nature of the file reception. This is clearly demonstrated by the results of the issue of determining how much resource is necessary to transmit incoming traffic at a certain level of quality.

Telecom operators should take the results obtained in this study into account when planning CIoT networks in order to avoid falling into unsatisfactory QoS situations.

## 8. Conclusions

In this paper, a model that takes into account the impulsive nature of IoT requests in CIoT networks was presented, and the performance of an LTE cell in the case of impulsive arrivals of NB-IoT requests was measured.

The impulse property of the arriving files significantly impacts solving the resource allocation problem. This effect should not be ignored because it may lead to a deterioration of system work and failure to achieve the required quality of service. This effect increases as the variance of the amount of group files increases.

The results we obtained in Section 7 prove and confirm the validity of the first and second research hypotheses.

The model proposed in this paper can provide a reference for determining the resources required to serve incoming traffic at different levels of impulsivity with specific performance indicators.

The limitations of the obtained results can be summed up as follows:

- In the proposed model, requests that do not find sufficient resources are considered lost requests without being stored in queues or buffers.
- In this model, the incoming traffic is also taken to be of a single nature.

Directions for further research are formulated by taking into consideration the above-given limitations:

- Performance evaluation study if requests that come in when the channel is busy are added to a queue or buffer. These requests wait in this buffer for a specified period and are then either served if the requested resources are available or blocked if the requested resources are not available.
- Study the heterogeneous nature of incoming traffic and develop the necessary models to evaluate the performance in this case.

## Figures and Tables

**Table 1:** Most common LPWAN technologies [10].

LPWAN Technologies	
Licensed Spectrum	Unlicensed Spectrum
NB-IoT	LoRaWAN
	Sigfox
LTE-M	NB-Fi
	RPMA
EC-GSM-IoT	СТРИЖ
	MIOTY
Thingstream	Helium
	SAT4M2M

**Table 2:** Summary of related works [author’s own processing].

References	Specifications	Focus Area	Contribution/Methodology	Limitations/Gaps
[9]	NB-IoT and LoRa	Resources management performance, challenges, and opportunities.	A comprehensive review of NB-IoT and an explanation of the most important challenges it faces.	Non-cellular LPWANs are not covered.
[12]	NB-IoT	Improvements were made in subsequent 3GPP releases, NB-IoT simulators, resource management, energy management, and applications.	A comprehensive survey of the research works conducted on various aspects of NB-IoT.	Only NB-IoT was studied.
[13]	eMTC and NB-IoT	Battery life time, latency, and maximum number of supported users.	eMTC and NB-IoT implementations in NS-3 in order to evaluate their performance.	The issue of resource management has not been studied.
[14]	NB-IoT	Performance evaluation of NB-IoT implementation in HetNet.	Comparing the average throughput and the energy consumption of NB-IoT in the cases of cooperative and non-cooperative resource allocation in 5G HetNet.	Impulsive traffic has not been studied.
[15]	NB-IoT	The case of NB-IoT with satellites.	Studying the performance of NB-IoT when implemented based on 5G technology via the Geostationary Equatorial Orbit (GEO).	There is no comparison with the performance of terrestrial networks.
[16]	NB-IoT	Limitations due to realistic channel estimation on NB-IoT performance.	The impact of coverage extension on the performance of NB-IoT terminals and the limitations that this event imposes on battery and communication delays were studied.	Only the uplink was studied.
[17]	NB-IoT	The device and network performance.	Evaluation of the experimental performance of NB-IoT and comparison with theoretical values.	Impulsive traffic has not been studied.
[18]	NB-IoT	The process of receiving and processing requests.	An improved strategy to schedule NB-IoT terminal servicing according to priority. Using Beta and Uniform distributions to model the arrival of resource requests.	The arrival of requests may be a random, impulsive arrival, and this limits the network performance.
[19]	NB-IoT	The impact of the NB-IoT network on system reliability.	Studying the effect of changing transmission distance and the presence of obstacles on the performance of smart systems based on NB-IoT.	Impulsive traffic has not been studied.
[20]	NB-IoT	Healthcare applications.	Measuring the performance of NB-IoT in health monitoring systems.	Only one use case was considered in this study.
[21]	NB-IoT and LTE	Smart power grid.	Developing a device that chooses between NB-IoT and LTE to transfer packets depending on the quality of the channels of each of these two technologies.	Only one use case was considered in this study.
[22]	LoRaWAN and Cellular NB-IoT	Power consumption, latency, security, and throughput.	An experimental comparison between the performance of NB-IoT and LoRaWAN.	In this study, the latest standards were not taken into consideration.
[23]	LoRaWAN, DASH7, and NB-IoT	The mobility, the connectivity, and the suitable technology for each use case.	An overview of the three technologies (LoRaWAN, DASH7, and NB-IoT) with a comparison between them.	The issue of resource management has not been studied.

**Table 3:** NB-IoT channels and signals [29].

Channel/Signal	Uplink/Downlink	Usage
Narrowband reference signal (NRS)	Downlink	Phase reference for downlink demodulation
Narrowband primary and secondary synchronization signals (NPSS and NSSS)		Synchronization in time and frequency
Narrowband physical broadcast channel (NPBCH)		Carries MIB
Narrowband physical downlink control channel (NPDCCH)		Control information
Narrowband physical downlink shared channel (NPDSCH)		Downlink data
Demodulation reference signal (DMRS)	Uplink	Reference for demodulation
Narrowband physical random access channel (NPRACH)		Random access
Narrowband Uplink Shared Channel (NPUSCH)		Uplink data and control information

**Table 4:** List of abbreviations (“author’s own processing”).

Abbreviation	Definition
3GPP	3rd Generation Partnership Project
5G	5th generation mobile network
BPSK	Binary phase-shift keying
CIoT	Cellular Internet of Things
CN	Core network
DL	Downlink
DMRS	Demodulation reference signal
DQPSK	Differential Quadrature Phase Shift Keying
DRX	Discontinuous Reception
eDRX	Extended Discontinuous Reception
eMTC	Enhanced Machine-Type Communication
eNB	Evolved Node B
FDD	Frequency Division Duplexing
GSM	Global System for Mobile communication
HetNet	Heterogeneous networks
IoT	Internet of Things
ISM	Industrial, Scientific and Medical band
LoRa	Long range
LoRaWAN	Long Range Wide Area Network
LoS	Line of sight
LPWA	Low-Power Wide-Area
LPWAN	Low Power, Wide Area Networks
LTE	Long Term Evolution
MIB	Master information block
NB	NarrowBand
NB-IoT	Narrowband Internet of Things
NOMA	Non-orthogonal multiple access
NPBCH	Narrowband physical broadcast channel
NPDCCH	Narrowband physical downlink control channel
NPDSCH	Narrowband physical downlink shared channel
NPRACH	Narrowband physical random access channel
NPSS	Narrowband primary synchronization signal
NPUSCH	Narrowband Uplink Shared Channel
NRS	Narrowband reference signal
NSSS	Narrowband secondary synchronization signal
NTN	Non-terrestrial network
OFDM	Orthogonal frequency-division multiplexing
PSM	Power saving mode
QoS	Quality of Service
QPSK	Quadrature Phase Shift Keying
SCFDMA	Single Carrier Frequency Division Multiple Access
UE	User Equipment
UL	Uplink

## Data Availability

Data is contained within this article.
